# 21-Benzylidene Digoxin: A Proapoptotic Cardenolide of Cancer Cells That Up-Regulates Na,K-ATPase and Epithelial Tight Junctions

**DOI:** 10.1371/journal.pone.0108776

**Published:** 2014-10-07

**Authors:** Sayonarah C. Rocha, Marco T. C. Pessoa, Luiza D. R. Neves, Silmara L. G. Alves, Luciana M. Silva, Herica L. Santos, Soraya M. F. Oliveira, Alex G. Taranto, Moacyr Comar, Isabella V. Gomes, Fabio V. Santos, Natasha Paixão, Luis E. M. Quintas, François Noël, Antonio F. Pereira, Ana C. S. C. Tessis, Natalia L. S. Gomes, Otacilio C. Moreira, Ruth Rincon-Heredia, Fernando P. Varotti, Gustavo Blanco, Jose A. F. P. Villar, Rubén G. Contreras, Leandro A. Barbosa

**Affiliations:** 1 Laboratório de Bioquímica Celular, Universidade Federal de São João del Rei, Campus Centro-Oeste Dona Lindú, Divinópolis, MG, Brazil; 2 Laboratório de Síntese Orgânica, Universidade Federal de São João del Rei, Campus Centro-Oeste Dona Lindú, Divinópolis, MG, Brazil; 3 Laboratório de Bioinformática, Universidade Federal de São João del Rei, Campus Centro-Oeste Dona Lindú, Divinópolis, MG, Brazil; 4 Laboratório de Biologia Celular e Mutagenicidade, Universidade Federal de São João del Rei, Campus Centro-Oeste Dona Lindú, Divinópolis, MG, Brazil; 5 Laboratório de Bioquímica de Parasitos, Universidade Federal de São João del Rei, Campus Centro-Oeste Dona Lindú, Divinópolis, MG, Brazil; 6 Laboratório de Biologia Celular e Inovação Biotecnológica, Fundação Ezequiel Dias, Belo Horizonte, MG, Brazil; 7 Laboratório de Farmacologia Bioquímica e Molecular, Instituto de Ciências Biomédicas, Universidade Federal do Rio de Janeiro, Rio de Janeiro, RJ, Brazil; 8 Laboratório de Bioquímica Microbiana, Instituto de Microbiologia Paulo Góes, Universidade Federal do Rio de Janeiro, Rio de Janeiro, RJ, Brazil; 9 Instituto Federal de Educação, Ciência e Tecnologia do Rio de Janeiro (IFRJ), Rio de Janeiro, RJ, Brazil; 10 Laboratório de Biologia Molecular e Doenças Endêmicas, Instituto Oswaldo Cruz/Fiocruz, Rio de Janeiro, RJ, Brazil; 11 Department of Physiology, Biophysics and Neurosciences, Center for Research and Advanced Studies (Cinvestav), Mexico City, Mexico; 12 Department of Molecular and Integrative Physiology, University of Kansas Medical Center, Kansas City, Kansas, United States of America; National Cancer Institute, United States of America

## Abstract

Cardiotonic steroids are used to treat heart failure and arrhythmia and have promising anticancer effects. The prototypic cardiotonic steroid ouabain may also be a hormone that modulates epithelial cell adhesion. Cardiotonic steroids consist of a steroid nucleus and a lactone ring, and their biological effects depend on the binding to their receptor, Na,K-ATPase, through which, they inhibit Na^+^ and K^+^ ion transport and activate of several intracellular signaling pathways. In this study, we added a styrene group to the lactone ring of the cardiotonic steroid digoxin, to obtain 21-benzylidene digoxin (21-BD), and investigated the effects of this synthetic cardiotonic steroid in different cell models. Molecular modeling indicates that 21-BD binds to its target Na,K-ATPase with low affinity, adopting a different pharmacophoric conformation when bound to its receptor than digoxin. Accordingly, 21-DB, at relatively high µM amounts inhibits the activity of Na,K-ATPase α_1_, but not α_2_ and α_3_ isoforms. In addition, 21-BD targets other proteins outside the Na,K-ATPase, inhibiting the multidrug exporter Pdr5p. When used on whole cells at low µM concentrations, 21-BD produces several effects, including: 1) up-regulation of Na,K-ATPase expression and activity in HeLa and RKO cancer cells, which is not found for digoxin, 2) cell specific changes in cell viability, reducing it in HeLa and RKO cancer cells, but increasing it in normal epithelial MDCK cells, which is different from the response to digoxin, and 3) changes in cell-cell interaction, altering the molecular composition of tight junctions and elevating transepithelial electrical resistance of MDCK monolayers, an effect previously found for ouabain. These results indicate that modification of the lactone ring of digoxin provides new properties to the compound, and shows that the structural change introduced could be used for the design of cardiotonic steroid with novel functions.

## Introduction

Cardiotonic steroids are common substances in the plant kingdom that confer a competitive advantage against depredation, either to the plant that synthesizes them, or to the animals that accumulates them from the diet [1]. Some experimental evidence shows that animals can endogenously synthesize cardiac steroids and that these substances play an important role in the regulation of blood pressure, cell proliferation and cell death [2], [3]. The basic structure of cardiotonic steroids consists of a steroid backbone with a cis/trans/cis configuration and a lactone moiety at position 17β, also known as the aglycone. In addition, these compounds frequently have a sugar attached at position 3β of the steroidal nucleus, for which they are commonly known as cardiac glycosides. The nature of the lactone ring distinguishes the cardenolides, that have an unsaturated butyrolactone ring, from the bufadienolides, which present an α-pyrone ring. The only structural difference between the two main cardenolides that are used therapeutically, digoxin and digitoxin, is a single extra hydroxyl group (–OH) on digoxin, which considerably alters its pharmacokinetics. Digitoxin, is more lipophilic, shows strong binding to plasma proteins, is almost entirely metabolized in the liver and exhibits a very long half-life. In contrast, digoxin displays weak binding to plasma proteins, is not extensively metabolized and is excreted in a primarily unaltered form by the kidneys [2], [3].

The main pharmacological effect of therapeutic doses of digoxin and digitoxin is their positive inotropic effect. This is mediated through the inhibition of the myocardial cell Na,K-ATPase, which results in an increase in the cytosolic levels of Na^+^ and secondarily Ca^2+^, due to reduction in transport of the Na^+^ dependent Na^+^/Ca^2+^ exchanger. The increased Ca^2+^ in the myocardial cell cytosol leads to further filling of the sarcoplasmic reticulum with Ca^2+^, which will be readily available for its release upon stimulation to produce enhanced muscle contraction [3]–[5].

In the context of cell biology, there are two areas in which cardiac steroids are of potential interest: cancer and cell adhesion. Both these phenomena are closely related, as shown by the loss of cell adhesion that takes place during cancer proliferation and metastasis. The effect of cardiac steroids on cell adhesion is poorly understood and is the focus of intense research [6]–[8]. Since the 1960s, several interesting antitumor effects have been observed for digitalis [9], [10], as well as for other cardenolides [11]–[15] and the related cardiac glycosides, the bufadienolides [16]–[19].

Human cancer predominantly affects epithelia [20], which cells are highly polarized and bound to each other through junctional complexes, including tight junctions (TJs). This last structure confers epithelia their capacity to function as effective barriers in the separation of biological compartments [21]. Loss of TJs is involved in cancer progression and metastasis [6], [22], for example, a decrease in the expression of the tight junction protein claudin (Cldn)-7, is involved in the dissemination of breast cancer cells [23]. Furthermore, the importance of Na,K-ATPase for the formation of junctional complexes has been well established [24], [25], as is the requirement of cell-cell contacts for the polarized expression of the Na,K-ATPase in lateral plasma membrane of epithelial cells [26]. Interestingly, the β_1_ subunit of Na,K-ATPase itself functions as a cell adhesion molecule in astrocytes [27] and epithelia [28]–[31].

A decrease in cell surface expression of the β_1_ subunit has been associated with epithelial-to-mesenchymal transition, a process involved in tumor invasiveness and metastasis [25], [32]. Treatment with several types of cardiac steroids causes various effects on cell junctions. High concentrations of ouabain (≥300 nM) and other cardiac steroids disrupt tight, adherens and communicating junctions as well as desmosomes of epithelial cells [24], [33], [34]. On the contrary, low concentrations of ouabain (10 nM) increase the sealing of TJs [35], accelerate cell polarity (as determined by the development of primary cilia [36]) and increase cell communication through communicating junctions [30].

Although studies on the biological effects of available cardiac steroids on tumor cells are rapidly developing, this is not the case for newly synthesized cardiotonic steroids. Some synthetic cardiotonic steroids have been tested for their anticancer activity [17], [37]–[44]. Oleandrin and 9-hydroxy-2″oxovoruscharin, a hemi-synthetic cardenolide, is under clinical trials and has demonstrated anticancer activity both *in* *vitro* and *in* *vivo*, in multiresistance cancer cells cultures [15], [43], [44]. Other studies demonstrated that several modifications of the sugar moiety of digoxin and digitoxin, can increase the anticancer effect of these compounds [45]–[48].

Several authors have described the synthesis of digoxin derivatives in which the addition of styrene groups to the lactone ring moiety produced some interesting biological activity [49], [50]. However, there are yet no reports concerning the anticancer activity or the effects of these digoxin derivatives on Na,K-ATPase activity.

The aim of this work was to study the biological effects of 21-benzylidene digoxin (21-BD), a digoxin derivative with an additional styrene group in the C21 carbon of the lactone ring, in cancer and normal cells.

## Materials and Methods

### General procedure for the synthesis of 21-benzylidene digoxin

Benzaldehyde (0.18 ml, 1.8 mmol), digoxin (0.469 g, 0.6 mmol), anhydrous K_2_CO_3_ (0.249 g, 1.8 mmol) and 60 ml of methanol were added into a round bottom flask. After stirring for 6 h at 70°C, the solvent was evaporated in a rotary evaporator. The crude product was diluted with 20 ml of water and extracted with hot ethyl acetate (3×30 ml). The organic layer was washed with brine, dried over anhydrous Na_2_SO_4_ and concentrated under vacuum. The crude product was then purified via silica column chromatography (CH_2_Cl_2_/MeOH 11∶1). After purification, the pure product was diluted in tetrahidrofurano (THF), precipitated with hexane and concentrated under reduced pressure to give 21-BD (0.325 g, 0.37 mmol, 62%) as a white solid.

### Cell culture

HeLa (human cervix carcinoma; ATCC CCL2) and RKO-AS45-1 (colon carcinoma; ATCC CRL-2579) cells were cultured in RPMI or DMEM/HAM F-10 (1∶1) medium (Sigma, St. Louis, MO, USA). CHO-K1 (ATCC CCL61) and MDCK-II cells (canine renal; ATCC CCL-34) were grown in DMEM. All media were supplemented with 10% fetal bovine serum (FBS; Hyclone) (CDMEM), 60 mg/ml streptomycin and 100 mg/ml penicillin. Cells were seeded at a density of 5×10^5^ cells/cm^2^ and incubated in a humidified atmosphere with 5% CO_2_. The culture medium was changed every 48 h to avoid nutrient depletion.

### Insect cell culture and viral infections

Sf-9 insect cells were grown in Grace’s medium with 3.3 g/l lactalbumin hydrolysate, 3.3 g/l yeastolate, and supplemented with 10% (v/v) fetal bovine serum, 100 units/ml penicillin, 100 µg/ml streptomycin and 0.25 µg/ml Fungizone. Cells were grown in suspension cultures and were transferred to 150 mm tissue culture plates before infection. Infections were performed as previously described [51]. After 72 h at 27°C, cells were scraped from the culture plates, centrifuged at 1,500×*g* for 10 min and suspended in 10 mM imidazole hydrochloride (pH 7.5) and 1 mM EGTA. Cells were homogenized on ice using a potter-Elvehjem homogenizer and the lysate was centrifuged for 10 min at 1,000×*g*. The supernatant was removed, centrifuged for an additional 10 min at 12,000×*g*, and the final pellet was suspended in 250 mM sucrose, 0.1 mM EGTA, and 25 mM imidazole HCl, pH 7.4.

### Cytotoxicity assay

Compound cytotoxicity effect was assessed using the MTT (3-(4,5-dimethylthiazol-2-yl)-2,5-diphenyltetrazolium bromide) tetrazolium salt colorimetric method (Sigma, St. Louis, MO, USA). Briefly, the cells were plated in 96-well plates (1×10^5^ cells/well) and incubated for 24 h at 37°C to confluence. Then, the wells were washed with culture medium and digoxin or 21-BD was added at different concentrations (0.05–500 µM). After incubation for 24 or 48 h, the plates were treated with MTT. Readings were performed in a Spectramax M5e microplate reader (Molecular Devices, Sunnyvale, CA, USA) at 550 nm. Cytotoxicity was scored as the percentage of reduction of absorbance, relative to untreated control cultures [52]. All experiments were performed in triplicate. The results were expressed as the mean of LC_50_ (the drug concentration that reduced cell viability to 50%).

### Apoptosis Assay

#### Comet assay

The single-cell gel electrophoresis assay (comet assay) was performed according to previously published protocols [53], [54]. For this, CHO-K1 cells were treated with 20, 35 or 50 µM digoxin or 21-DB, which are concentrations that do not affect cell viability according to the MTT assay. The cells were seeded in 24-well plates. The next day cells were washed twice with PBS, and incubated 3 h with the corresponding compounds in culture media without serum. The negative and positive control groups were treated with PBS, and methyl methanesulfonate (400 µM), respectively. After 24 h, the cells were washed twice with PBS and detached using a trypsin-EDTA solution. Trypsin was inactivated with 3.0 ml of complete medium, followed by centrifugation (5 min, 150×*g*). The pellet was then resuspended in 500 µl of PBS, and 30 µl aliquots of the cell suspensions were mixed with 70 µl of low melting point agarose (0.5%). These mixtures were placed on slides pre-coated with normal melting point agarose (1.5%) and covered with coverslips. The coverslips were removed after five minutes, and the slides were immersed overnight in lysis solution (NaCl 2.5 M, EDTA 100 mM, Tris 10 mM, pH 10; Triton X-100 1% and DMSO 10%). Next, slides were washed with PBS and maintained for 40 min in a horizontal electrophoresis box filled with cold alkaline buffer (EDTA 1 mM, NaOH 300 mM, pH>13). Electrophoresis was conducted at 0.86 V/cm and 300 mA (20 minutes) and the slides were subsequently neutralized (0.4 M de Tris, pH 7.5), fixed with methanol and stained with ethidium bromide. Visual analyses were performed under fluorescence [55], [56].

#### Phosphatidylserine translocation

HeLa cells were plated in 60 mm diameter Petri dishes at confluence and incubated overnight in CDMEM. They were then treated with 2 µM digoxin or 50 µM 21-BD in CDMEM for 6, 12 or 24 h. After incubation, suspended cells were recovered from the media by centrifugation at 150×*g*, 20°C for 5 minutes. Attached cells were obtained by a mild protease treatment 1 ml Accutase plus 3 ml PBS), centrifuged as indicated above and added to the cells obtained from the media. Annexin-V bound to the outer leaflet of the plasma membrane was measured with a comercial kit (Annexin-V-Fluos Stainig kit, Roche, Germany) following manufacturer instructions. Briefly, cell suspension was incubated with a mixture of Annexin-F-FITC and propidium iodide in saline buffer for 15 min. Cell suspension was analyzed by flow cytometry (FACSCalibur flow cytometer, Fow Jo software).

### Transepithelial electrical resistance (TER)

MDCK cells were cultured on Transwell permeable supports (3415, Corning Inc., NY, USA) the transepithelial electrical resistance was measured with an EVOM and the EndOhm-6 system (World Precision Instruments, Sarasota, FL, USA). Final values were obtained by subtracting the resistance of the bathing solution and the empty insert. The results are expressed in ohms·cm^2^ (Ω·cm^2^) as a percentage of the control.

### Immunofluorescence

After TER measurements, MDCK monolayers were washed three times with ice-cold PBS/Ca^2+^, fixed with 4% paraformaldehyde for 30 min at 4°C, permeabilized with 0.1% Triton X-100 for 5 min, blocked for 30 min with 3% BSA and treated for 1 h at 37°C with a specific primary antibody. The monolayers were then rinsed 3 times with PBS/Ca^2+^, incubated with an appropriate FITC or TRICT-labeled antibodies for 30 min at room temperature and rinsed as indicated above. Filters with the cells were excised with a scalpel and mounted in Vectashield (Vector Labs, Burlingame, CA, USA). The preparations were examined with a Leica confocal SP5 microscope (Leica Microsystems, Wetzlar, Germany). The captured images were imported into FIJI, version 2.8 (National Institutes of Health, Baltimore, USA), to obtain maximum projections and into the GNU Image Manipulation Program (GIMP) to normalize brightness and contrast in all images and construct figures.

### Immunoblotting

Western blot analysis of whole-cell protein extracts was performed as described previously [57]. Briefly, MDCK and HeLa cells were washed with PBS and solubilized with radioimmunoprecipitation assay (RIPA) buffer [10 mM piperazine-*N*,*N’*-bis(2-ethanesulfonic acid), pH 7.4, 150 mM NaCl, 2 mM ethylenediamine-tetraacetic acid (EDTA), 1% Triton X-100, 0.5% sodium deoxicholate, and 10% glycerol] containing protease inhibitors (Complete Mini; Roche Diagnostics, Indianapolis, IN). The protein content of the cell lysate was measured (BCA protein assay reagent; Pierce Chemical, Rockford, IL) and prepared for SDS-polyacrylamide gel electrophoresis (PAGE) by boiling in sample buffer. The resolved proteins were electrotransfered to a polyvinylidene difluoride membrane (Hybond-P; GE Healthcare, Little Chalfont, Buckinghamshire, United Kingdom). The proteins of interest were then detected with the specific polyclonal or monoclonal antibodies indicated in each case, followed by species-appropriate peroxidase-conjugated antibodies (Zymed Laboratories, South San Francisco, CA) and chemiluminescent detection (ECL PLUS; GE Healthcare).

### Detection of Na,K-ATPase and claudin expression via real-time quantitative RT-PCR

For real-time quantitative RT-PCR (RT-qPCR), total RNA was extracted from the cell samples using TRIzol (Life technologies, USA). The concentration was estimated via spectrophotometry with a Nanodrop ND2000 (Thermo Scientific, USA). All reverse transcriptase reactions were performed using 5 µg of RNA with the Superscript III kit (Invitrogen, USA) according to the manufacturer’s instructions. All RNA samples were reverse transcribed simultaneously to minimize the inter assay variation associated with the reverse transcription reaction. Real-time quantitative PCR was performed on an ABI Prism 7500 fast sequence detection system (Applied Biosystems) using Go Taq qPCR master mix (Promega, USA). The following primers and concentrations were employed (Murphy et Al, 2004): Na,K-ATPase α_1_ subunit (GenBank accession number NM_000701): Fw (300 nM): 5′-TGTCCAGAATTGCAGGTCTTTG-3, Rv (300 nM): 5′-TGCCCGCTTAAGAATAGGTAGGT-3′; Na,K-ATPase β_1_ subunit (GenBank accession number NM_001677): Fw (300 nM): 5′-ACC AAT CTT ACC ATG GAC ACT GAA-3′, Rv (300 nM): 5′-CGG TCT TTC TCA CTG TAC CCA AT-3′; Cldn-2 Fw GGTGGGCATGAGATGCACT, Rv CACCACCGCCAGTCTGTCTT; Cldn-4 Fw TGCACCAACTGCGTGGAGGATGAG, Rv ACCACCAGCGGGTTGTAGAAGTCC. The applied PCR conditions were as follows: 50°C for 2 minutes, followed by 40 cycles at 95°C for 15 sec and 60°C for 1 min. Each of these primer sets generated a unique PCR product, as confirmed by the obtained melting curves. The PCR assays were performed in triplicate, and the data were pooled. Relative quantitative measurement of target gene levels was performed using the ΔΔCt method of Livak et al. [58]. As endogenous housekeeping control genes, we employed the glyceraldehyde 3-phosphate dehydrogenase (GAPDH, GenBank accession number NM_002046) and ribosomal 18S subunit genes (GenBank accession number NR_003286) [59]. The following primers and concentrations were used: GAPDH Fw (300 nM): 5′- ATGTTCGTCATGGGTGTGAA-3′, GAPDH Rv (300 nM): 5′-GGTGCTAAGCAGTTGGTGGT-3′, 18S Fw (300 nM): 5′-CAGCCACCCGAGATTGAGCA-3′, 18S Rv (300 nM): 5′-TAGTAGCGACGGGCGGTGTG-3′.

### Molecular modeling analyses

**Figure 1:**
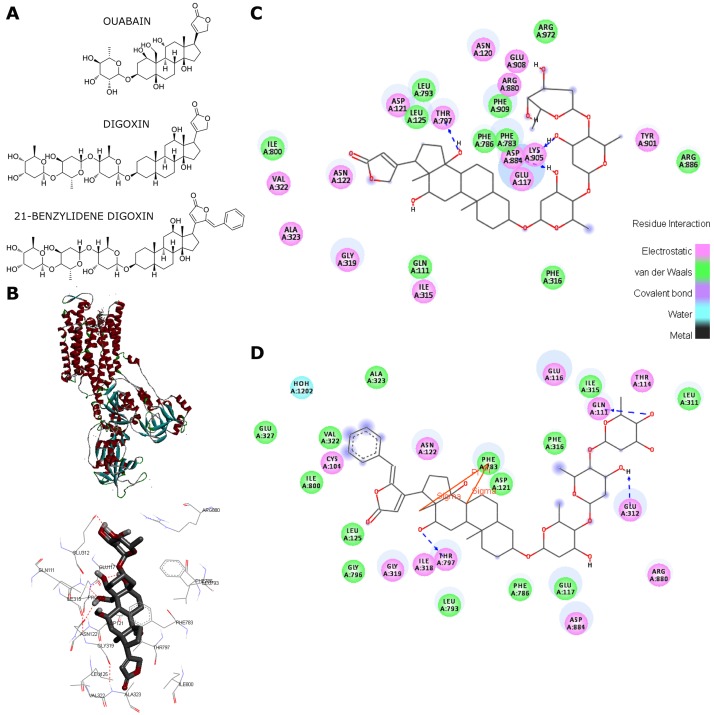
Structure and pharmacophoric conformation of 21-BD. (A) Chemical structure of ouabain, digoxin, and 21-BD. (B) Left: whole structure of Na,K-ATPase (PDB: 4HYT) showed in solid ribbon representation, where the alpha-helix, beta-sheets and turns are in red, blue and gray, respectively; right: highlight of the binding site with ouabain shown in tube representation. Dashed red lines indicate hydrogen bonds. Only polar hydrogen atoms were showed for a better visualization. Pharmacophoric conformation of, (C) digoxin and (D) 21-BD; polar and nonpolar interactions are depicted by magenta and green colors, respectively. Dashed lines indicate hydrogen bonds. Residue interactions are color coded as indicated in the inserted scale.

Initially, digoxin, ouabain, and 21-BD (Figure 1A) were generated and refined via the semi-empirical PM6 method [60] implemented in Gaussian 09 W software [61]. The crystallographic structure of Na,K-ATPase (target protein) complexed with ouabain was obtained from the Protein Data Bank (PDB ID: 4HYT [62] with a 3.40 Å resolution, using the α_1_ subunit of Na,K-ATPase). The magnesium ion and three interstitial water molecules were kept in the binding site. Next, a rigid re-dock of ouabain was carried out to validate our system. Subsequently, digoxin and 21-BD were docked against the ATPase. The docking analyses were conducted using AutoDock Vina 1.0.2 [63]. The applied search algorithm was Iterated Local Search Global Optimizer for global optimization. In this process, a succession of steps with mutation and local optimization (the Broyden-Fletcher-Goldfarb-Shanno [BFGS] method) were conducted, and each step followed the Metropolis criterion [64]. In this study, a grid box was constructed exploring all active sites, which was defined as a cube with the geometric center in ouabain, with dimensions of 20×20×24 Å, spaced points of 1 Å and X, Y and Z coordinates of −27.065, 20.469 and −69.469, respectively. All molecular modeling figures were constructed using DS Visualizer 3.1 [65].

### Preparation of Pdr5p plasma membranes

Plasma membranes containing the overexpressed Pdr5p protein were prepared from the *Saccharomyces cerevisiae* mutant strain AD124567 as previously reported [66].

### Fractionation of cell lysates and preparation of membrane fractions

A total of 2.5×10^5^ cells were grown in 75 cm^2^ culture bottles and treated with digoxin and 21-BD. After 48 hours of treatment, cells were washed three times with cold PBS and scrapped from the culture bottle with a rubber policeman in a membrane preparation buffer (6 mM Tris [pH 6.8], 20 mM imidazole, 0.25 M Sucrose, 0.01% SDS, 3 mM EDTA and 2 mM PMSF). The cells were homogenized in a potter-Elvehjem homogenizer, using ten stokes in ice. Then, they were sonicated in an ultrasonic cell disruptor on ice for 10 s at 45% power. The sample was subjected to centrifugation at 20,000×g for 90 min at 4°C. The supernatant was discarded and the pellet resuspended in 250 µl of membrane preparation buffer. Finally, this sample was sonicated for 10 s at 25% power until complete homogenization.

### Na,K-ATPase preparation from rat brain hemispheres

Brain hemispheres from adult male Wistar rats were rapidly collected after diethylether anesthesia and decapitation. The protocols used for the use of rats were approved by the Institutional Commission for Ethics in the Use of Animals, process code DFBCICB011 and conformed to the Guide for the Care and Use of Laboratory Animals, published by US National Institute of Health (NIH publication No. 85-23, revised in 1996). The brain tissues, used as a source of ouabain-sensitive (α_2_/α_3_) Na,K-ATPase isoforms, were homogenized in 250 mM buffered sucrose, 2 mM dithiothreitol, 0.1 mM PMSF and 5 mM Tris/HCl (pH 7.4) with a motor-driven Teflon Potter-Elvehjem homogenizer. Subsequently, chaotropic treatment with 2 M KI was performed for 1 h under constant stirring, followed by centrifugation three times at 100,000×*g* for 1 h. The final pellet was resuspended in 250 mM sucrose, 0.1% sodium deoxycholate and 20 mM maleate/Tris (pH 7.4), then stored overnight at −20°C and subjected to differential centrifugation after thawing [67]. The pellets were resuspended in the same buffer without PMSF and stored in liquid N_2_.

### Na,K-ATPase preparation from mouse kidney

Kidney tissue was isolated from adult mice and was homogenized in 250 mM sucrose, 0.1 mM EGTA, and 25 mM imidazole HCl, pH 7.4, using a motor-driven Teflon Potter*-*Elvehjem homogenizer. The sample was then subjected to centrifugation at 4,500×*g* for 10 min. The resulting supernatant was centrifuged at 70,000×*g* for 1 h. The final pellet was resuspended in the homogenization solution and used for Na,K-ATPase activity assays. All experimental protocols involving mice were approved by the University of Kansas Medical Center Institutional Animal Care and Use Committee.

### NTPase Assays

Enzymatic activities were assayed using ATP as a substrate in standard medium (50 µl final volume) containing 100 mM Tris-HCl pH 7.5, 4 mM MgCl_2_, 75 mM KNO_3_, 7.5 mM NaN_3_, and 0.3 mM ammonium molybdate in the presence of 3 mM ATP. The reaction was initiated by the addition of 13 µg/ml of the plasma membrane preparations, then maintained at 37°C for 60 min and stopped by the addition of 1% SDS, as previously described [68]. The released inorganic phosphate (Pi) was measured as described elsewhere [69]. Using stock solutions in DMSO, 21-BD and digoxin were added up to a 5% v/v final concentration. The difference in ATPase activity in the presence or absence of 3 µM oligomycin corresponded to a Pdr5p-mediated ATPase activity.

### Measurement of the effect of 21-BD on Na,K-ATPase activity

The brain hemisphere preparations were incubated at 37°C for 2 h in medium containing 87.6 mM NaCl, 3 mM KCl, 3 mM MgCl_2_, 3 mM ATPNa_2_, 1 mM EGTA, 10 mM sodium azide and 20 mM maleic acid/Tris (pH 7.4), and the cell membrane preparations were incubated at 37°C for 1 h in 120 mM NaCl, 20 mM KCl, 2 mM MgCl_2_, 3 mM ATPNa_2_ and 50 mM HEPES, (pH 7.5), both in the absence and presence of 1 mM ouabain or increasing concentrations of 21-BD and digoxin. Na,K-ATPase activity was determined by measuring the Pi released according to a colorimetric method described previously [69], and specific activity was considered as the difference between the total and ouabain-resistant ATPase activities [70].

### Ouabain binding

HeLa cells were plated on 24 multi well plates at confluence and cultured overnight. Monolayers were then rapidly washed three times with potassium free saline buffer (140 mM NaCl, 1.8 mM CaCl_2_, 5 mM sucrose, 10 mM Tris-HCl pH 7.4 at room temperature) and incubated 30 min with total binding solution (0.1×10^−6^ ^3^H-ouabain plus 0.9×10^−6^ cold ouabain in potassium free saline buffer) under gentle agitation and at room temperature. Then monolayers were washed four times, 1 min each, with ice cold 0.1 M MgCl_2_ and dissolved with 400 µl of SDS 1%. ^3^H activity was measured in samples of 350 µl by scintillation counting. Total ^3^H-ouabain binding was competed by adding to the total binding solution the necessary amount of 21-BD to reach the concentrations indicated in results. A subset of monolayers was exposed to competed binding solution (total binding solution plus 0.5×10^−4^ M cold ouabain) to measure the unspecific binding and subtract it to the total binding values.

### Statistical analyses

Statistical analyses were performed with GraphPad Prism software, version 5. The results were expressed as mean ± error standard of the mean. Statistical significance in a one-way analysis of variance (ANOVA), followed by a Bonferroni’s selected pairs comparison test, was set at *P*<0.05 (*), *P*<0.01 (**), or *P*<0.001 (***) vs. the control condition, and “n” represents the number of independent experiments.

## Results

We synthesized 21-BD via a simple stereo selective vinylogous aldol reaction, according to Xu et al. [50], and characterized the final product through conventional NMR, HRMS and IR analysis (Figure 1A; Figure S1 and Data S1).

To perform the molecular modeling analyses we started with the geometric optimization of ligands, through a semi-empirical approach, to correct geometric parameters such as bond lengths and refine the structure. The re-dock structure obtained with the software Autodock Vina, shows that the refined and the crystallographic ligand share the same conformation at the active site, with a root mean square deviation value of 2.24 Å for the best solution, which indicated the accuracy of the methodology used (see Figure S2A). Figure 1B shows a simulation of the docking of ouabain to its binding site in the Na,K-ATPase α_1_ isoform. As shown, the enzyme preserves the secondary structural elements of the ATPase family (Figure 1B, left) and that the active site is composed of Gln111, Glu117, Pro118, Asn122, Leu125, Glu312, Ile315, Gly319, Val322, Ala323, Phe783, Phe786, Leu793, Ile800, Arg880 (Figure 1B, right), in agreement with known structures [71].

Following these molecular modeling analyses we docked ouabain, digoxin and 21-BD to Na,K-ATPase (Figure S2B and Figure 1C and D). Simulations resulted in binding energies for ouabain, digoxin, and 21-BD of −9.8, −1.9, and −10.0 kcal/mol, respectively. These data suggest that 21-BD may have a similar binding affinity to the Na,K-ATPase than ouabain and digoxin. However, both these last compounds exhibit different pharmacophoric conformations in the binding site, principally at the level of the lactone ring. Figures 1C and D highlight the most important intermolecular interactions between the ligands and the target protein. Digoxin binds to the enzyme via hydrogen bonding with Thr797, Asp884 and Lys905 aminoacids. The electrostatic and hydrophobic interaction occur with Glu117, Asn120, Asp121, Asn122, Ile315, Gly319, Val322, Ala323, Arg880, Asp884, Tyr901, Glu908 and Gln111, Leu125, Phe316, Phe783, Phe786, Leu793, Arg886, Phe909, Arg972, respectively (Figure 1C). In contrast to natural cardiac glycosides, 21-BD complexes to the Na,K-ATPase through Gln111, Glu312, and Thr797 hydrogen bonding. In addition, the aromatic ring reaches a hydrophobic pocket formed principally by Cys104, Val322, Ala323, Glu327, Ile800 (Figure 1D). Moreover, the pharmacophoric conformation is completely different from that of the natural glycosides, whose aromatic moiety binds to water molecules and magnesium ion.

**Figure 2:**
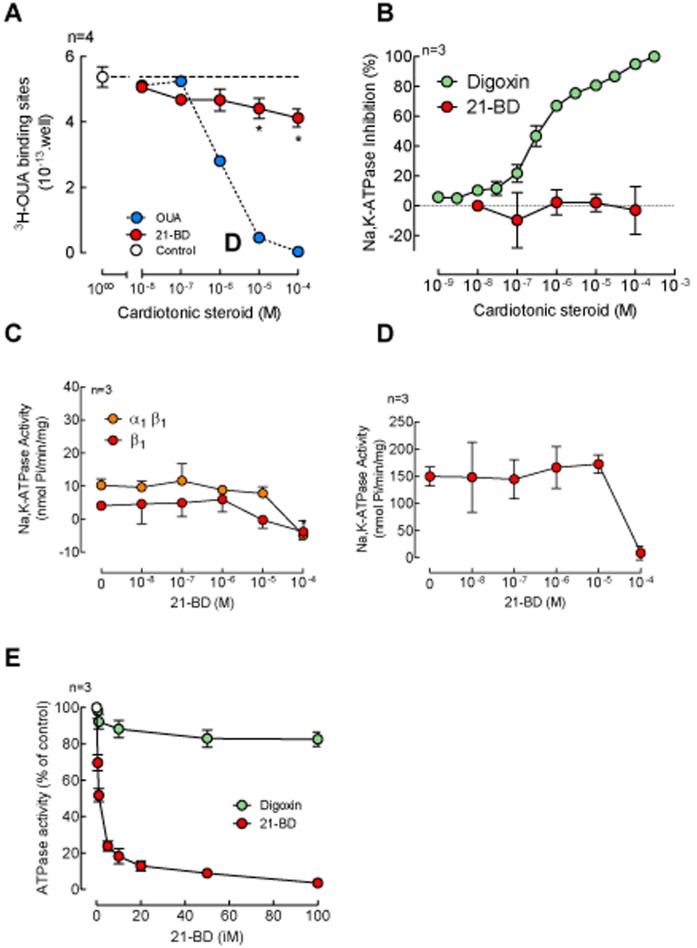
21-BD effect on Na,K-ATPase and Pdr5p activity. (A) 21-BD competition of ^3^H-ouabain binding on HeLa cells; the control for maximal binding is represented with a white circle and a long dashed line, competition of ouabain and 21-BD is shown with blue and red circles respectively. (B) Inhibition of rat´s brain hemisphere Na,K-ATPase after 2 h incubation with digoxin (green circles) or 21-BD (red circles). (C) Effect of 21-BD on the Na,K-ATPase activity on proteins expressed in Sf9 insect cells, Na,K-ATPase activity was measured on Sf9 cells expressing the rat α_1_ β_1_ (orange circles) or β_1_ (red circles) after 15 min treatment with the indicated concentrations of 21-BD. (D) Dose-response curve for the effects of 21-DB on Na,K-ATPase activity of mouse kidney membrane preparations. E) Effect of 21-BD (red circles) or digoxin (green circles) on the activity of the Pdr5p transporter.

To directly determine binding of 21-BD to the ouabain site of the Na,K-ATPase, we first tested if the synthetic steroid could compete with ^3^H-ouabain binding in HeLa cells, which express the α_1_ isoform of the Na,K-ATPase [72]. As shown in Figure 2A, 21-BD significantly competed with ouabain binding at concentrations in the µM range, indicating that the affinity of the Na,K-ATPase for this ligand is low. We next studied if 21-BD affects Na,K-ATPase activity of a membrane preparation from rat cerebral hemispheres. This tissue is mainly composed of the ouabain sensitive α_2_ and α_3_ isoforms of the enzyme, which comprise approximately 80% of the total Na,K-ATPase activity, with the remaining 20% corresponding to the α_1_ isoform [73]. Digoxin inhibited most of the Na,K-ATPase activity, with an IC_50_ of 219±40 nM, corresponding to the high-affinity sites of the α_2_ and α_3_ isoforms, and at higher concentrations it further inhibited the α_1_ isoform. In contrast, 21-BD had no effect on rat brain Na,K-ATPase, even at the highest concentration tested (100 µM) (Figure 2B).

To further determine if 21-BD had any effect on the α_1_ isoform of the Na,K-ATPase, we tested its activity of two different sources of Na,K-ATPase rich in α_1_. We used membrane fractions from Sf9 insect cells exogenously expressing the rat Na,K-ATPase α_1_ and β_1_ subunits and membrane fractions from mouse kidney, which primarily contains the Na,K-ATPase α_1_ isoform. The expression in Sf9 cells provides a useful system to specifically investigate effects on the exogenously expressed Na,K-ATPase, since these cells virtually lack expression of an endogenous Na,K-ATPase. As shown in Fig. 2C–D, 21-BD inhibited activity of α_1_β_1_ from Sf9 cells, but had little effect on parallel preparations expressing only the β_1_ subunit of the enzyme, which by lacking the catalytic α_1_ subunit serve as a control. The effect of 21-BD only took place at high concentrations (100 µM) of the compound. Similarly, 21-BD inhibited the mouse kidney Na,K-ATPase with similar kinetics. These results agree with the ^3^H-ouabain binding experiments in HeLa cells, suggesting that 21-BD interacts with Na,K-ATPase with low affinity.

Although the results described above suggest that Na,K-ATPase α_1_ is a receptor for 21-BD, the possibility exists that 21-BD could be also targeting another protein/s in the membrane of the cells. Steroids have been shown to inhibit the activity of yeast and mammalian ATP-dependent efflux pumps [74], [75]. Therefore, we investigated the effect of 21-BD on Pdr5p, a member of the *Saccharomyces cerevisiae* ABC transporters family that shares many substrates and inhibitors with the mammalian P-glycoprotein [76]. Interestingly, after incubation with membrane preparations from *Saccharomyces cerevisiae*, 21-BD showed a concentration-dependent inhibitory effect on NTPase activity, with an IC_50_ of 1.25±0.36 µM. In contrast, digoxin had no significant effect on Pdr5p (Figure 2E). This suggests that, while 21-BD has an effect on its Na,K-ATPase target, it can also affect other proteins, such as the ATP dependent transporter of the plasma membrane.

**Figure 3:**
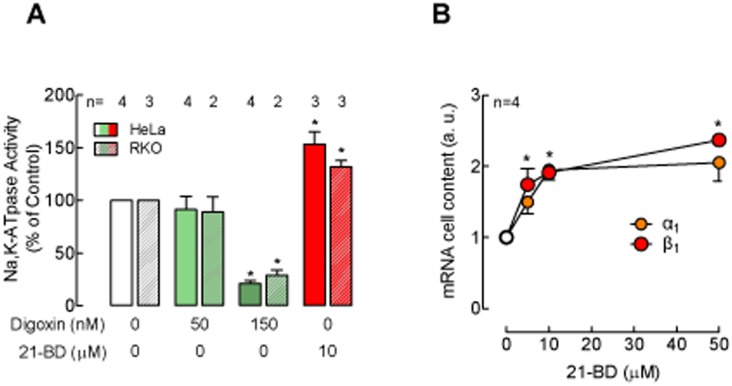
21-BD increases Na,K-ATPase expression in cancer cells. (A) Na,K-ATPase activity after incubation of HeLa cells with 21-BD or digoxin for 48 h with different concentrations of 21-BD. B) mRNA content of the Na,K-ATPase α_1_ and β_1_ subunits of HeLa cells after 48 h incubation with various concentrations of 21-BD.

To further study the effects of 21-BD, its action was tested and compared to that of digoxin in whole cells. Thus, the compounds were directly applied to HeLa cervix cells, which express the Na,K-ATPase α_1_ isoform, and RKO colorectal cancer cells that, in addition to α_1_, express the α_3_ isoform [77]. After incubation with the compounds for 48 h, cells were harvested and Na,K-ATPase activity determined. As expected, treatment with 150 nM digoxin for 48 h inhibited Na,K-ATPase activity (Figure 3A, green columns). Surprisingly, 10 µM 21-BD increased Na,K-ATPase activity in both cell lines (Figure 3A, red columns). To explore whether the increase in Na,K-ATPase activity was caused by an increase in expression of the Na,K-ATPase, the levels of Na,K-ATPase subunits in the cells was determined by RT-PCR. As depicted if Figure 3B, incubation with 10 µM 21-BD for 48 h increased mRNA of the Na,K-ATPase α_1_ and β_1_ subunits in HeLa cells.

**Figure 4:**
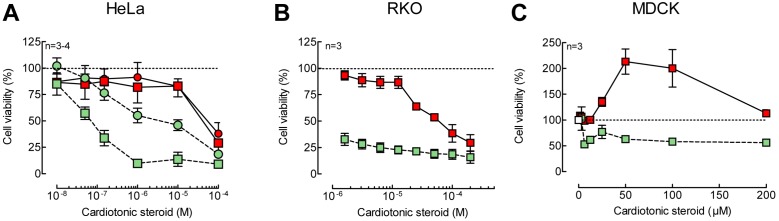
High concentrations of 21-BD reduce cell viability of HeLa and RKO. HeLa (A) or RKO (B) cells were treated with digoxin (green symbols) or 21-BD (red symbols) for 24 (circles) or 48 (squares) h. Viability was measured by MTT reduction assay. 100 and 25 µM 21-BD induced the statistically significant reduction of HeLa and RKO viability, respectively (p<0.0084). Digoxin reduces HeLa viability starting with 150 µM for 24 h and 50 µM for 48 h (p<0.001). RKO cells have a higher sensitivity to digoxin that induces statistically significant differences starting from 1.6 µM for 48 h (p<0.0001).

To test the possibility that 21-BD could exhibit anticancer effects, such as those described for digoxin, we used HeLa, RKO cancer cells, and normal epithelial MDCK cells. Both digoxin and 21-BD showed a cytotoxic effect on HeLa cells after treatment for 24 and 48 h. Digoxin produced the classical time- and dose-dependent decrease in viability (LC_50_ of 2.2±0.8 µM), being approximately 25 times more potent that 21-BD (LC_50_ of 56,16±8,12 µM), (Figure 4A). In RKO cells, digoxin decreased viability with higher potency, with an LC_50_ of 0.42±0.1 µM, compared to that of 21-BD, which had a LC_50_ of 55.81±15.15 µM. Thus, while RKO cells showed a higher sensitivity to digoxin than HeLa cells (Figure 4B), they exhibited the same sensitivity to 21-BD than HeLa cells. Surprisingly, different from digoxin, 21-BD increased the viability of MDCK cells (Figure 4C). This unexpected result may depend on the fact that 21-BD is not toxic to MDCK cells and that it may induce a higher mitochondrial activity to metabolize the substrate in the MTT assay.

**Figure 5:**
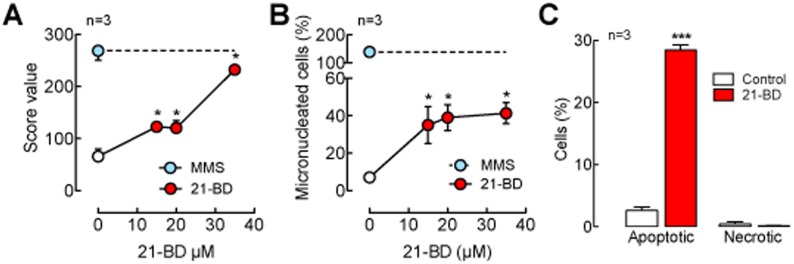
21-BD induces apoptosis in HeLa and CHO-K1 cells. (A) Score value obtained from the comet assay of CHO-K1 cells incubated 24 h with 21-BD at different concentrations (red circles). (B) Micronucleated cells percentage of CHO-K1 cultures incubated with 21-BD at different concentrations for 24 h. A 24 h incubation with 0.4 mM Methyl methanesulfonate (MMS) was used as a control (A, B, blue circles). (C) Apoptotic and necrotic HeLa cells after 24 h of incubation in control media (white bars), media with 50 µM 21-BD (red bars) or 2 µM digoxin (green bars) for 24 h. Apoptosis and necrosis were detected by flow cytometry ussing an annexin-V translocation assay and the incorporation of propidium iodide in to the nucleus, respectively. *P*<0.01.

Digoxin is known to induce apoptosis in several cell types [78], [79]. Therefore, the effect of 21-BD in reducing HeLa and RKO cell viability could be due to induction of cell apoptosis. To assess this possibility, we followed two crucial events of the apoptotic process after 21-BD treatment: DNA fragmentation and the translocation of phosphatidylserine from the inner towards the outer leaflet of the plasma membrane of CHO-K1 cells. As shown in Figures 5A and B, 21-BD causes primary DNA fragmentation as determined by comet assay. The amount of phosphatidylserine translocated was measured through the binding of fluoresceinated annexin-V and the amount of necrotic cells was estimated by the incorporation of propidium iodide into the cell nucleus (Figure 5C). We found that 21-BD increases phosphatidylserine translocation (red bar, Apoptotic) but not necrosis (Necrotic, red bar). Altogether, these results show that 21-BD is able to induce cell apoptosis.

**Figure 6:**
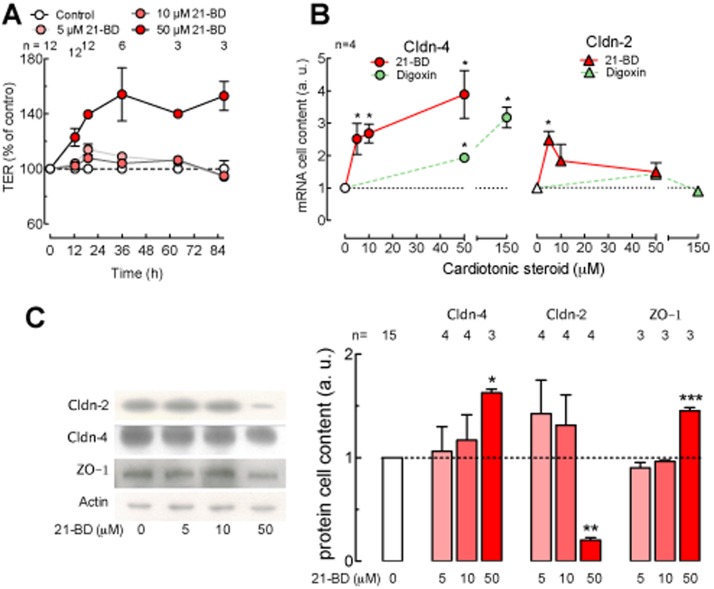
21-BD regulates tight junctions. MDCK cells were cultured in transwell permeable supports and treated with 5, 10 and 50 µM 21-BD. (A) TER was measured as a function of time. The control TER data (white circles, dotted line) averaged 183±8 Ω.cm^2^ (n = 13) and were normalized to 100%. 5 and 10 µM 21-BD provoke transient small increases of TER, while 50 µM 21-BD causes a stronger and a sustained TER increase (red circles). (B) MDCK cells were incubated 48 h with different concentrations of 21-BD (red symbols) or digoxin (green symbols). mRNA cell content of claudins -4 (circles) and -2 (triangles) were measured by quantitative real time PCR. (C) Protein cell content of the tight junction integral membrane proteins claudins -4 and -2 and the membrane-associated protein ZO-1 as a function of 21-BD concentration in the media for 48 h. Images from the left part of the figure C are representative immunoblots and the graph in the right part is the statistical analysis.

The integrity of cell junctions is a key factor in cancer biology, as several authors have demonstrated that alterations of tight junctions are associated with cell transformation and metastasis [6], [23], [41], [80], [81]. We tested whether 21-BD treatment modifies transepithelial electrical resistance (TER) or the distribution of the tight junction proteins Cldn-2 and -4 and ZO-1. For this purpose, we used MDCK cells, which are a well-known model for tight junction studies [82]. We first examined whether 21-BD altered TER. As shown in Figure 6A, 50 µM 21-BD induces a sustained increase in TER for at least 87 h of treatment and in a dose-dependent manner. This change in TER is associated with an increase in the expression of tight junction proteins. Figure 6B shows that 21-BD increases the cellular content of claudin-4 mRNA at all concentrations tested (Figure 6B, red circles) and claudin-2 mRNA only at the lowest concentration (Figure 6B, red triangles). Conversely, digoxin increases claudin-4 (Figure 6B, green circles) but not claudin-2 mRNA (Figure 6B, green triangles). The increase in claudin-4 mRNA results in a corresponding increment of the protein at high 21-BD concentrations (Figure 6C) while claudin-2 protein strongly decreases (Figure 6C, Cldn-2, red bars). 21-BD also induces the increment of ZO-1, an important peripheral membrane protein of tight junctions (Figure 6C).

**Figure 7:**
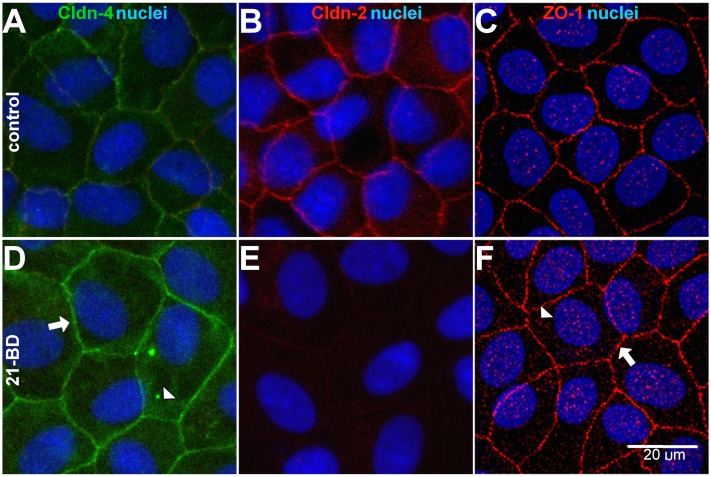
21-BD regulates tight junctiońs proteins localization. Confluent monolayers of MDCK cells, grown on filters, were maintained in control medium (A, B, C) or treated with 50 µM 21-BD for 48 h (D, F, G) and processed for immunofluorescence, using antibodies against the TJs proteins: the integral membrane proteins claudin-4 (Cldn-4, A, D green) and claudin-2 (Cldcn-2, B, E red) and the peripheral membrane protein ZO-1 (C, E, red). Nuclei were stained with TOPRO (blue). 21-BD increases claudin-4 and ZO-1 expression at the tight junction (arrows) and in the cytoplasma (arrow heads), while simultaneously reduces the expression of claudin-2.

Figure 7 illustrates that 21-BD increases the expression of claudin-4 and ZO-1 at the tight junction (arrows) and the cytoplasm of MDCK cells (arrow heads D and F vs A and C), displaying their characteristic “chicken fence” like pattern of expression of these junctional proteins. Conversely, 21-BD reduces the expression of claudin-2 (Figure 7E vs B). The coordinated antagonistic variation of claudins -4 and -2 has also been shown in epithelial cells treated with other factors, e.g. EGF [83], [84].

**Figure 8:**
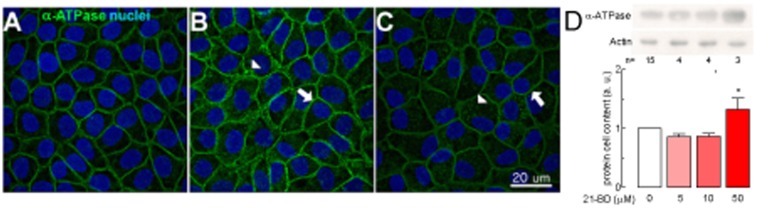
21-BD increases the expression of Na,K-ATPase in MDCK cells. (A) Protein cell content of the α_1_ subunit of the Na,K-ATPase of confluent monolayers of MDCK cells grown on filters in control medium (white bar) or treated with different concentrations of 21-BD (red bars) for 48 h; upper part of the figure A shows representative immunoblots of the α_1_ subunit of the Na,K-ATPase and actin, the lower part the densitometric analysis. (B and C) Na,K-ATPase α_1_ subunit stained with a fluoresceinated antibody (B, C, white) or Topro (blue) to detect the nuclei.

21-BD also increases the cellular content of the α_1_ Na,K-ATPase at the highest concentration tested (Figure 8A) and its localization in the plasma membrane (Figure 8C, arrow) and in the cytoplasm (Figure 8C, arrow heads) of MDCK cells.

## Discussion

We have synthetized a digoxin derivative, 21-BD, that reduces cell viability by inducing apoptosis, and increases the hermeticity of tight junctions through the up-regulation of claudin-4 and ZO-1 and the down-regulation of claudin-2 junctional proteins. These effects take place after 12 to 48 h incubation with 21-BD. These delayed responses most probably result from the activation of signaling cascades that result in changes in expression of different genes, as has been reported for other cardenolides [85]–[87]. An important question is whether 21-BD triggers those effects as a result of its binding to the Na,K-ATPase or to another membrane protein. 21-BD is able to displaces ^3^H-ouabain from the Na,K-ATPase (Figure 2A), demonstrating that 21-BD has the capacity to bind to the Na,K-ATPase with a low affinity and that 21-BD occupancy of the sites in the pump may trigger cellular responses via the classical Na,K-ATPase mediated cascade of intracellular mediators. Molecular modeling also indicates the binding of 21-BD to the α-Na,K-ATPase (Figure 1D), although with a pharmacophoric conformation very different from that of digoxin. Also, supporting the role of the Na,K-ATPase as a 21-BD receptor is the regulation that this cardiotonic steroid exerts on the tight junctions in MDCK cells, which is similar to that caused by ouabain [35]. In addition, 21-BD induces apoptosis like other cardiotonic steroids do. Nevertheless, it is known that adrenocortical bovine cells express high affinity binding sites for ouabain that are distinct from the Na,K-ATPase [88]. Here we show that 21-BD, but not digoxin, is able to inhibit the activity of the yeast ABC ATPase Pdr5p (Figure 2E), suggesting the possibility that 21-BD might, in addition to the Na,K-ATPase, act through a different receptor.

Our docking results show that the aromatic ring of 21-BD may reach a hydrophobic pocket in the binding site (Figure 1), suggesting that intermolecular hydrophobic interactions may be optimized by linking alkyl groups to the aromatic ring or by increasing the aliphatic chain between lactone and aromatic rings. In other words, extending the molecule in the direction of the hydrophobic pocket may improve the affinity of new compounds through extra van der Waals interactions with the enzyme. Additionally, the docking simulations revealed a large change in the pharmacophoric conformation of 21-DB at the lactone ring, which may explain the biological effect of this compound.

Retrospective clinical observations performed 40 years ago suggest that cardiac steroids have anticancer effects [10], [89], [90]; however, few studies have been performed to elucidate the underlying mechanisms of these effects [3], [4]. It has been proposed that cardiotonic steroids induce necrosis through the inhibition of the ion transport activity of the Na,K-ATPase, the resulting sustained increase of the cytosolic calcium concentration and the activation of cellular signaling cascades [16], [91]–[94]. Nevertheless, low concentrations of ouabain (10 nM) do not induce apoptosis in MDCK cells [35], while high concentrations (300 nM - 3 µM) induce necrotic processes such as cell swelling independent of Na,K-ATPase pumping activity [95] and permeabilization of lysosomal membranes [42]. These findings indicate that cardiotonic steroids induce cell death though complex mechanisms named oncosis, a type of cell death that involves apoptotic (caspase activation) as well as necrotic (cell swelling) processes [95]. Our data indicate that 21-BD induces DNA damage, which is a signature characteristic of apoptosis but is also compatible with oncosis. *In vitro* studies show that other mechanisms may be related to cell responses triggered by cardiac glycosides. For instance, bufalin, a hydrophobic cardiotonic steroid, downregulates the expression of Cyclin A, Bcl-2 and Bcl-X_L_ and upregulates the expression of p21 and Bax in ovarian endometrial cyst stromal cells, affecting cell cycle progression and inducing apoptosis [96]. Digoxin applied at low concentrations (<10 nM) prevents apoptosis in HeLa cells, whereas at higher concentrations induces the release of cytochrome-c, thereby triggering apoptosis [97]. UNBS1450 induces death in A549 lung cancer cells through the NFκB signaling pathway [42]. The diversity of reported effects indicates the need for further studies aimed to elucidate the possible mechanisms involved in cell death induced by digoxin and other types of cardiac steroids.

21-BD is moderately cytotoxic for HeLa and RKO cells (LC_50_, of approximately 50 µM). This cytotoxicity is correlated with the induction of DNA damage. Studies addressing the genotoxicity of cardiac glycosides are scarce [98], [99]. Given that cardiac glycosides produce reactive oxygen species (ROS) [4], [100] and that these substances are well-known genotoxic agents [101], it is possible that 21-BD could induce an increase in ROS. Alternatively, 21-BD may inhibit topoisomerase II, as is known for other cardiac glycosides, including digoxin [91].

When directly applied to HeLa and RKO cells and in relatively low concentrations, 21-BD induces the increase of Na,K-ATPase activity through up-regulation of the α_1_ and β_1_ subunits of the enzyme (Figures 3A, B). In contrast, in relatively high concentrations, 21-BD inhibits the activity of the Na,K-ATPase α_1_ isoform of cell homogenates (Fig. 2C, D). This effect appears to be isoform specific, since 21-DB has no effect on the α2 and α_3_ isoforms of brain that have a high affinity for for cardiac steroids. The requirement of high concentrations of 21-DB to inhibit α_1_ shows the low affinity of this Na,K-ATPase isoform for the compound. The effects that we observe with lower concentrations of 21-BD are consistent with the activation of the signaling functions of the Na,K-ATPase that are known to trigger a variety of cellular phenomena including induction of endocytosis of junctional proteins [34], [83] and the Na,K-ATPase itself [102]–[104], the detachment of epithelial cells from the substrate and from themselves [24], [105], cell proliferation [106], protection from cell death caused by the addition of serum [107], cell survival [108] and the sealing of tight junction through the upregulation of claudins [35]. The effects produced by 21-BD depend on the cellular context. While this compound reduces cell viability and induces apoptosis in cells derived from tumors (HeLa and RKO), it does not affect the viability of normal kidney epithelial cells (MDCK) as indicated by the increase in the transepithelial electrical resistance. The overexpression of claudin and the strengthening of tight junctions is an important mechanism against the phenotypic characteristics of cancer, which can regulate events such cell migration, transformation, proliferation and invasiveness [109]–[113].

Our results suggests that the modification of lactone ring of digoxin and maybe other cardiotonic steroids, could be an interesting alternative for chemical modification of this class of compounds. The effects presented by 21-BD appear to be unrelated with the classical effects that the cardiotonic steroids exert on intracellular ion changes through changes in Na,K-ATPase activity and raises the possibility that 21-BD (and maybe other cardiotonic steroids) may be involved with other proteins different from the Na,K-ATPase. Interestingly, digoxin has been shown to bind to other proteins besides Na,K-ATPase, such as the retinoic acid receptor-related orphan nuclear receptor RORγt. Digoxin-21-salicylidene is another digoxin derivative, with similar modification than 21-BD, that does not have high cytotoxic effect and selectively binds to RORγt, reducing progression of autoimmune encephalitis [49]. So, the decrease of cardiotonic steroid cytotoxicity opens the opportunity to use this class of compounds in other diseases.

Surprisingly, Na,K-ATPase is not the only enzyme that is responsive to 21-BD. Although Na,K-ATPase activity was not directly affected by 21-BD, the activity of the yeast multidrug exporter Pdr5p was largely inhibited by 21-BD. Cholesterol-derived steroids can block the pumping function of this transporter, showing an IC_50_ in the micromolar range, and some derivatives are considered interesting leads for use as drugs for the putative treatment of multidrug-resistant (MDR) tumors [74], [75]. It is also known that several cardiotonic steroids exhibit anticancer activity in cells that express the chemotherapy resistance phenotype [43], [44], [114]–[117]. This activity may be explained if as suggested by our 21-BD results, these cardiotonic steroids, have also the capacity to inhibit MDR proteins. Alternatively, anti MDR activity may be caused by downregulation of c-Myc or MDR-related genes, the inhibition of the glycosylation of MDR-related proteins or the inhibition of glycolysis [43]. Our results show, for the first time, that a cis-trans-cis steroid is also able to cause direct inhibition of Pdr5p activity and opens the possibility that 21-BD may act as a drug to reverse the MDR phenotype.

Another important action of 21-BD is its interaction with junctional complexes. This increases TER as a result of the upregulation of the tight junction proteins Claudin-4 and ZO-1, which increase the sealing of tight junctions [118]–[120], and downregulates the levels of Claudin-2, which is expressed in epithelia with low TER [121], [122]. Additionally, 21-BD induces an increase in the levels of Claudin-4 mRNA. This effect shows that ouabain is not the sole cardiotonic steroid that alters the molecular composition of tight junctions and TER [24], [35], but that 21-BD can induce these effects as well.

## Conclusion

The combination of an styrene group with the lactone moiety of digoxin produced a cardiotonic steroid, 21-BD, that enhances Na,K-ATPase catalytic activity in intact cells and stimulates Na,K-ATPase mediated signaling events. The later include reduction of cell viability via apoptosis in cancer, but not normal epithelial cells and increase in the closure of tight junctions of epithelial cells. The chemical modification of cardiotonic steroids with the styrene group has the potential to provide compounds with new functional properties that could results beneficial to treat cancer and perhaps other diseases in which cell proliferation occurs.

## Supporting Information

Figure S1 **Synthesis of 21-BD.**
(TIF)

Figure S2 **Structure and pharmacophoric conformation of ouabain.** (A) Crystallographic (green) and docked (red) structures of ouabain. (B) Pharmacophoric conformation of ouabain. Ouabain complexes with the receptor via hydrogen bonding between Gln111, Glu117, Asp121, Asn122, Glu312 and Thr797. In addition, eletrostatic and hydrophobic interactions perform around the steroid core composed by Pro118, Leu125, Gly319, Phe783, Phe786, Leu793, Ile800, Arg880 and Asp121, Ile315, Val322, Ala323, respectively.
(TIF)

Data S1 **Spestroscopic characterization of 21-BD.**
(DOC)
