# Supplementary figures and images for: 21-Benzylidene Digoxin: A Proapoptotic Cardenolide of Cancer Cells That Up-Regulates Na,K-ATPase and Epithelial Tight Junctions
Source: PLoS One. 2014 Oct 7;9(10):e108776. doi: 10.1371/journal.pone.0108776 (PMC4188576; doi:10.1371/journal.pone.0108776)

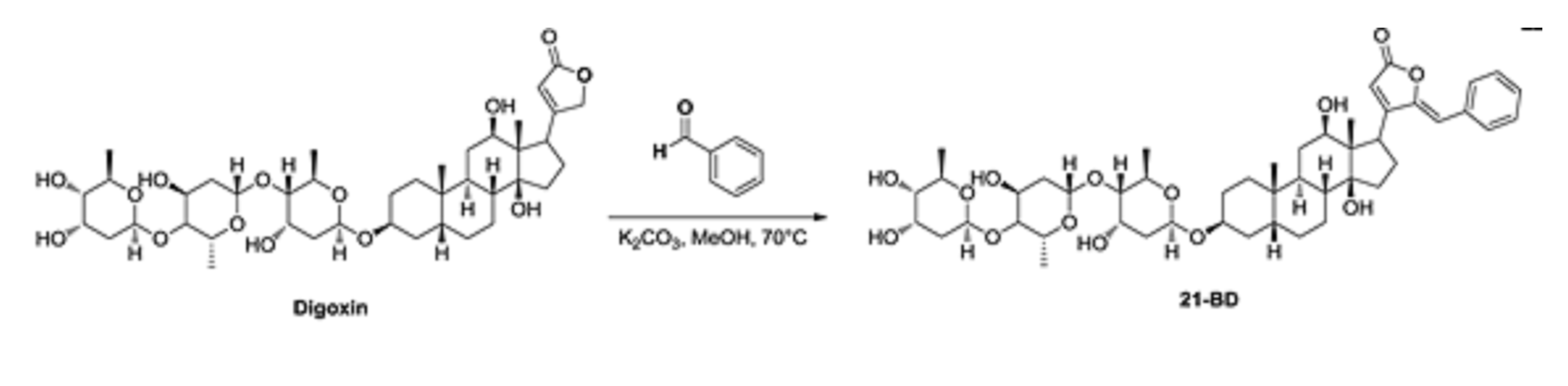

Supplement: Figure S1 — Synthesis of 21-BD. (TIF) [file pone.0108776.s001.tif]

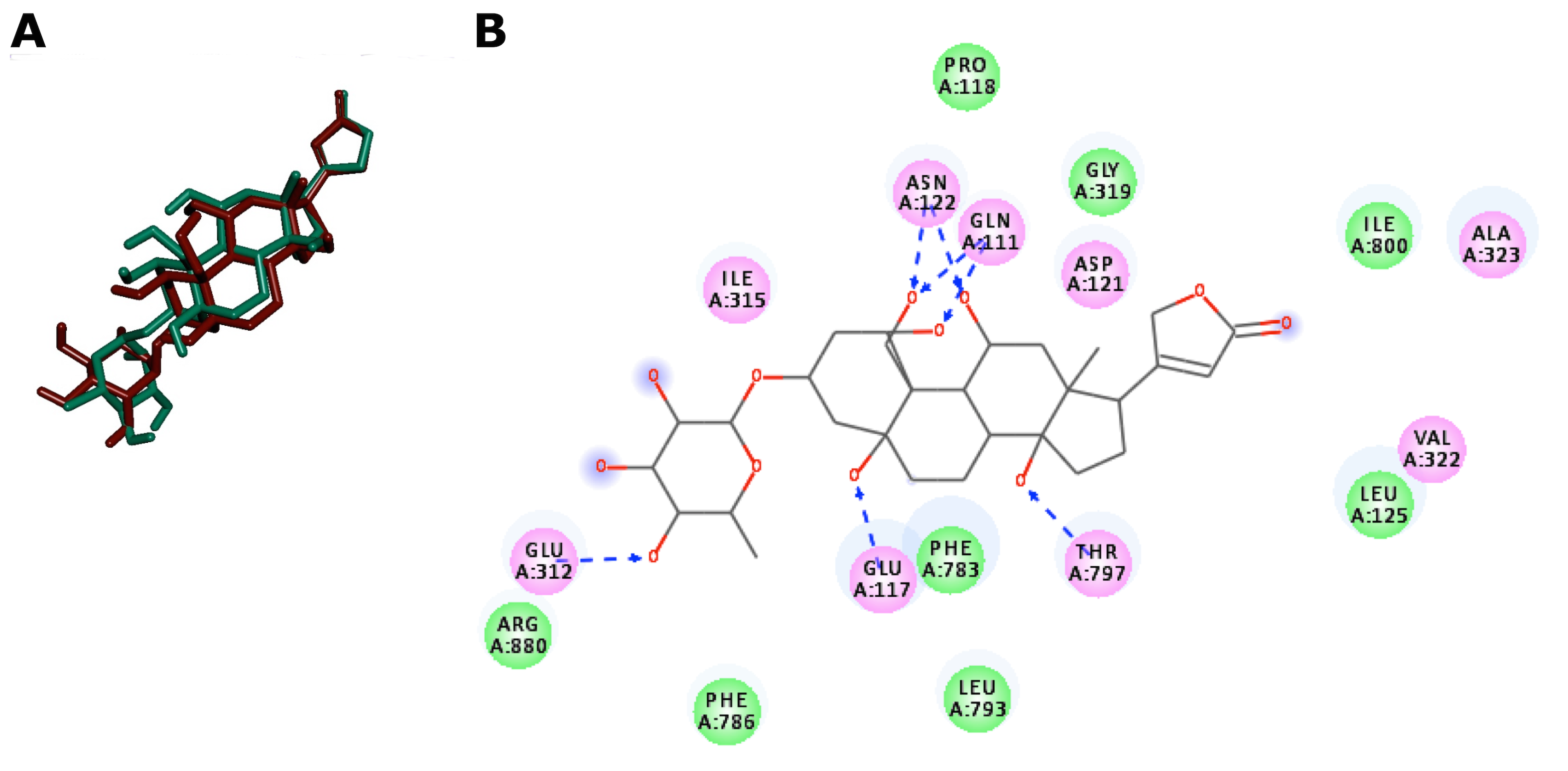

Supplement: Figure S2 — Structure and pharmacophoric conformation of ouabain. (A) Crystallographic (green) and docked (red) structures of ouabain. (B) Pharmacophoric conformation of ouabain. Ouabain complexes with the receptor via hydrogen bonding between Gln111, Glu117, Asp121, Asn122, Glu312 and Thr797. In addition, eletrostatic and hydrophobic interactions perform around the steroid core composed by Pro118, Leu125, Gly319, Phe783, Phe786, Leu793, Ile800, Arg880 and Asp121, Ile315, Val322, Ala323, respectively. (TIF) [file pone.0108776.s002.tif]
