# Supplementary material for: 21-Benzylidene Digoxin: A Proapoptotic Cardenolide of Cancer Cells That Up-Regulates Na,K-ATPase and Epithelial Tight Junctions
Source: PLoS One. 2014 Oct 7;9(10):e108776. doi: 10.1371/journal.pone.0108776 (PMC4188576; doi:10.1371/journal.pone.0108776)
Supplement: Data S1 — Spestroscopic characterization of 21-BD. (DOC) [file pone.0108776.s003.doc]

**Spestroscopic characterization of 21-BD**

**^1^H NMR** (400 MHz, CDCl_3_) δ (ppm): 0.66 (s, 3H); 0.85 (s, 3H); 1.06-1.15 (m, 9H); 1.25-1.9 (m, 25H); 3.00 (ddd, *J* = 2.94 Hz, *J* = 6.87 Hz, *J* = 9.54 Hz, 2H); 3.11-3.15 (m, 3H); 3.61-3.75 (m, 5H); 3.84-3.93 (m, 3H); 4.20 (d, *J* = 2.16 Hz, 1H); 4.26 (d, *J* = 2.16 Hz, 1H); 4.59-4.63 (m, 3H); 4.79-4.83 (m, 3H); 5.00 (d, *J* = 5.47 Hz, 1H);6.20 (s, 1H); 6.76 (s, 1H), 7.36-7.38 (m, 1H); 7.45 (t, *J* = 7.56 Hz, 2H); 7.67-7.70 (m, 2H).

**^13^C NMR** (100 MHz, CDCl_3_) δ (ppm): 169.1, 166.4, 149.7, 133.0, 130.0, 128.8, 128.6, 114.7, 110.0, 98.9, 98.8, 95.1, 84.9, 81.7, 81.5, 73.5, 72.5, 71.9, 68.9, 67.4, 67.3, 66.8, 66.1, 66.0, 55.6, 41.8, 40.3, 38.2, 38.1, 37.7, 36.2, 34.5, 32.2, 31.5, 30.0, 29.8, 29.4, 28.8, 26.3, 25.9, 23.5, 21.2, 18.2, 17.9, 9.2.

**IV** (υ_max_, KBr, cm^-1^): 3462, 1695, 1603, 1499.

**HRMS** (m/z): [M+Na]^+^ 891.4505 (Calculated C_48_H_68_NaO_14_^+^ = 891,4501).
